# Physical activity and risk of atrial fibrillation in the general population: meta-analysis of 23 cohort studies involving about 2 million participants

**DOI:** 10.1007/s10654-020-00714-4

**Published:** 2021-01-25

**Authors:** Setor K. Kunutsor, Samuel Seidu, Timo H. Mäkikallio, Richard S. Dey, Jari A. Laukkanen

**Affiliations:** 1 National Institute for Health Research Bristol Biomedical Research Centre, University Hospitals Bristol and Weston NHS Foundation Trust and University of Bristol, Bristol, UK; 2 Musculoskeletal Research Unit, Translational Health Sciences, Bristol Medical School, University of Bristol, Learning & Research Building (Level 1), Southmead Hospital, Bristol, BS10 5NB UK; 3 Leicester Diabetes Centre, Leicester General Hospital, Gwendolen Road, Leicester, LE5 4WP UK; 4 Diabetes Research Centre, Leicester General Hospital, University of Leicester, Gwendolen Road, Leicester, LE5 4WP UK; 5 Division of Cardiology, Department of Internal Medicine, Oulu University Hospital, Oulu, Finland; 6 University of Ghana Hospital, Legon, Ghana; 7 Institute of Public Health and Clinical Nutrition, University of Eastern Finland, Kuopio, Finland; 8 Faculty of Sport and Health Sciences, University of Jyväskylä, Jyväskylä, Finland; 9 Department of Medicine, Central Finland Health Care District, Jyväskylä, Finland

**Keywords:** Physical activity, Cardiac arrhythmias, Atrial fibrillation, Cohort study, Risk factor, Systematic review, Meta-analysis

## Abstract

Regular physical activity is well established to be associated with reduced risk of cardiovascular disease outcomes. Whether physical activity is associated with the future risk of atrial fibrillation (AF) remains a controversy. Using a systematic review and meta-analysis of published observational cohort studies in general populations with at least one-year of follow-up, we aimed to evaluate the association between regular physical activity and the risk of AF. Relevant studies were sought from inception until October 2020 in MEDLINE, Embase, Web of Science, and manual search of relevant articles. Extracted relative risks (RRs) with 95% confidence intervals (CIs) for the maximum versus the minimal amount of physical activity groups were pooled using random-effects meta-analysis. Quality of the evidence was assessed by GRADE. A total of 23 unique observational cohort studies comprising of 1,930,725 participants and 45,839 AF cases were eligible. The pooled multivariable-adjusted RR (95% CI) for AF comparing the most physically active versus the least physically active groups was 0.99 (0.93–1.05). This association was modified by sex: an increased risk was observed in men: 1.20 (1.02–1.42), with a decreased risk in women: 0.91 (0.84–0.99). The quality of the evidence ranged from low to moderate. Pooled observational cohort studies suggest that the absence of associations reported between regular physical activity and AF risk in previous general population studies and their aggregate analyses could be driven by a sex-specific difference in the associations – an increased risk in men and a decreased risk in women.

**Systematic review registration:** PROSPERO 2020: CRD42020172814

**Supplementary information:**

The online version contains supplementary material available at (10.1007/s10654-020-00714-4).

## Introduction

Cardiac arrhythmias, which are cardiovascular outcomes, are cardiac rhythm disorders that constitute a public health burden. Atrial fibrillation (AF) is the most common arrhythmia worldwide and is associated with increased morbidity and mortality and reduced quality of life [1, 2]. Structural heart diseases, such as coronary heart disease (CHD), cardiomyopathy, and valvular disease, are the strongest risk factors for developing AF [3]. Regular physical activity has several health benefits and it is well established to reduce the risk of cardiovascular disease (CVD) outcomes [4–6]. Coronary heart disease is the most typical manifestation of CVD. Given that CHD and AF share some common risk factors and the well-known inverse relationship between physical activity and CHD [6, 7], one would expect that physical activity might have a beneficial effect on AF. Over the last decades, several reports have been published on the associations between physical activity and the risk of AF in the general population, but the results have been divergent; with some studies reporting decreased AF risk with regular physical activity[8–10] and others reporting an increased AF risk [11, 12] or no evidence of an association. [13, 14] Several meta-analyses have also been conducted in efforts to pool the existing evidence, but findings of these earlier reports have also been inconsistent. Whereas some reviews have found no significant association between regular physical activity and AF, [15, 16] others have suggested a reduced risk of AF with regular physical activity, [17–19] which makes the topic very controversial given the persisting uncertainty. Furthermore, some of these previous meta-analyses included studies of competitive athletes. Also, they pooled the results of case–control and/or cross-sectional study designs in addition to those of observational cohort designs [17, 19]. Case–control study designs are characterised by substantial selection bias and lack temporality between exposure and outcome; hence the results of some of these reviews may be misleading. Furthermore, in contrast to the potential beneficial effects of regular physical activity on risk of AF, some reports have suggested that physical activity may increase the risk of AF in the general population. [11, 12] Since the publication of the last relevant review, [17] several studies evaluating the association between physical activity and risk of AF have been published. Due to the controversial nature of this topic, we sought to evaluate in detail the prospective nature, magnitude and specificity of the association between physical activity and risk of AF in the general population (non-athletes) using a systematic review and meta-analysis of all observational cohort studies published to date.

## Methods

### Data sources and searches

We registered this systematic review and meta-analysis in the PROSPERO prospective register of systematic reviews (CRD42020172814). It was based on a predefined protocol and conducted per PRISMA and MOOSE guidelines [20, 21] (Appendix 1–2). MEDLINE and Embase were searched from inception to 23 October 2020 with no restriction on language. The computer-based searches used a combination of keywords or terms relating to physical activity and arrhythmias (atrial fibrillation). Full details of the search strategy are presented in Appendix 3. Titles and abstracts of retrieved citations were initially screened by one author (SKK) to assess their suitability for potential inclusion, followed by the acquisition of full texts for detailed evaluation. Full-text evaluation was independently conducted by two authors (SKK and SS) with discussion with a third author (JAL) to reach consensus when there were disagreements. The reference lists of key studies and review articles were manually scanned for additional studies and citing references were also checked in Web of Science.

### Study selection

We included observational population-based observational cohort (retrospective or prospective, case-cohort, or nested case–control) studies if they had at least 1 year of follow-up and examined the relationship of physical activity with the risk of first AF in adult general populations. The following studies were excluded: (i) case–control study designs; (ii) those based on athletes and/or evaluated competitive or endurance sports; and (iii) those evaluating the associations between measures of fitness (eg, cardiorespiratory fitness, exercise capacity) and risk of AF.

### Data extraction and quality assessment

One author (SKK) initially extracted data from eligible studies using a predesigned data collection form and a second author (SS) independently checked the data with that in original articles. Disagreements were discussed with the involvement of a third author (JAL). Data were extracted on the following study characteristics: author and year of publication, geographical location, year of enrolment, study design, demographic characteristics (age, sex), sample size, duration of follow-up, assessment of physical activity, number of outcome events, and the most fully-adjusted relative risks (RRs), hazard ratios (HRs), or odds ratios (ORs) of outcomes (and corresponding 95% confidence interval [CIs]). When there were multiple publications involving the same cohort, study selection was limited to a single set of most comprehensive results to avoid double counting of a cohort in the pooled analysis. The critical factor used for selection was the most up-to-date comprehensive study (most extended follow-up or analysis covering the largest number of participants). The risk of bias within individual observational studies was assessed using the Cochrane Risk of Bias in Non-randomised Studies—of Interventions (ROBINS-I) tool. [22] This tool assesses risk of bias for confounding, participant selection, classification of interventions, deviations from intended interventions, missing data, outcome measurements and selective reporting. Risk is quantified in each domain as low risk, moderate risk, serious risk or critical risk, then an overall judgement of the risk of bias is provided for each study. We also used the Grading of Recommendations Assessment, Development and Evaluation (GRADE) approach to assess the quality of the body of evidence, based on study limitations, inconsistency of effect, imprecision, indirectness and publication bias [23].

### Data synthesis and analysis

Summary measures of association were reported as RRs with 95% CIs. Except for one study [24], all studies categorised physical activity exposure (e.g., leisure-time physical activity, intensity of physical activity, or total or any physical activity) into two or more groups. Risk estimates could not be transformed to consistent comparisons (e.g., top versus bottom thirds of the distribution of physical activity) using standard statistical methods previously described [25, 26] because of the varying reporting of outcomes. However, to enhance comparison and interpretation of the findings, the extreme groups (i.e., top versus bottom or maximum versus the minimal amount of physical activity) were used for the analyses. This approach, which we have utilised in a previous review [27] is considered reliable as we have shown that pooled estimates from transformed and untransformed data are qualitatively similar [28]. For the study that reported the risk comparison as a continuous measure (per standard deviation (SD) change) [24], this was transformed to a top versus bottom quantile using standard statistical methods [29] described previously. [25, 27] When a study assessed specific types of physical activity in addition to total or any physical activity, we only used risk estimates for total or any physical activity in the pooled analysis. For studies that reported estimates of the association, according to subgroups (e.g., by sex), we obtained a within-study summary estimate using a fixed effect meta-analysis. Relative risks were pooled using a random effects model to minimize the effect of heterogeneity [30]. Standard chi-square tests and the I^2^ statistic were used to quantify the extent of statistical heterogeneity across studies.[31, 32] To determine the degree of heterogeneity, we also estimated 95% prediction intervals, which provide a region in which about 95% of the true effects of a new study are expected to be found [33, 34]. Pre-specified study-level characteristics such as geographical location, sex, the average age at baseline, body mass index (BMI), the average duration of follow-up (< 12 vs. ≥ 12 years based on the distribution of the data and average range of follow-up), number of cases, and study quality were explored as sources of heterogeneity, using stratified analysis and random effects meta-regression [35]. To evaluate small study effects, we visually inspected constructed Begg’s funnel plots [36] and performed Egger’s regression symmetry test [37]. All analyses were conducted using Stata version MP 16 (Stata Corp, College Station, Texas).

## Results

### Study identification and selection

**Fig. 1:**
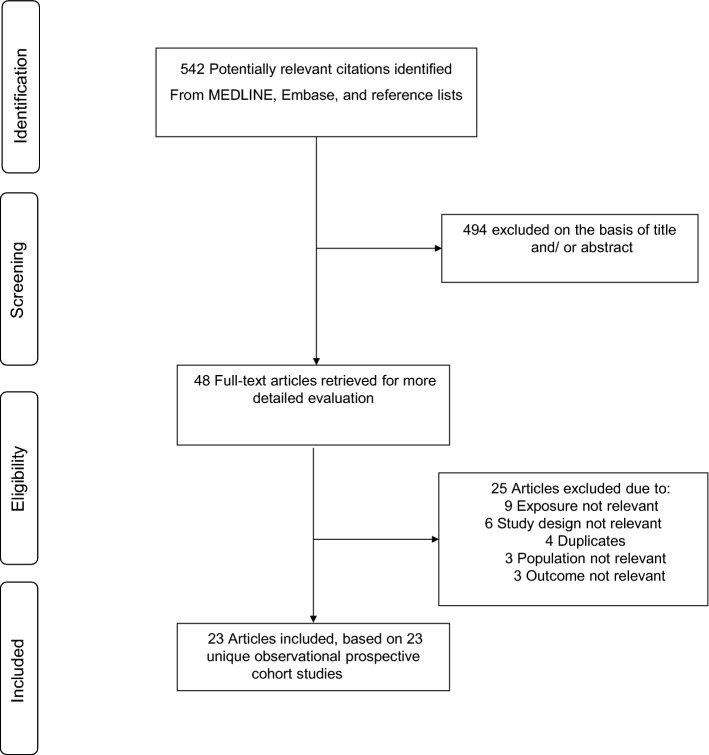
PRISMA flow diagram

The study selection process is illustrated in Fig. 1. Our search of databases and manual screening of relevant articles identified 542 potentially relevant citations. Following the screening of titles and abstracts, 48 articles remained for full-text evaluation. We reviewed and excluded 25 articles because (1) exposure was not relevant (n = 9); (2) study design not relevant (n = 6); (3) they duplicated a previous publication using the same cohort (n = 4); (4) population not relevant (n = 3) and (5) outcome not relevant (n = 3). In total, we included 23 articles representing 23 unique observational cohort studies comprising of 1,930,725 general population participants and 45,839 AF events.[8–13, 24, 38–53]

## Study characteristics and quality

Table 1 summarises the characteristics of the eligible studies evaluating the associations between physical activity and AF. Except for two studies based on retrospective cohort designs, all studies were based on prospective cohort study designs. Publication years ranged between 2005 and 2020. All 23 studies reported on AF outcomes, with one study additionally reporting data on VAs [8]. For studies providing these data, the average age and BMI of participants at baseline ranged from approximately 38–73 years and 23.7–29.0 kg/m^2^, respectively; the weighted means (SDs) were 49.9 (7.9) years and 25.1 (1.6) kg/m^2^ respectively. Three studies enrolled women only, three only men, and the rest enrolled both males and females. Ten studies were based in Europe (Denmark, the Netherlands, Norway, Sweden and UK), eight in North America (USA), four in Asia (Japan and Korea) and one in the Pacific (Australia). The average duration of follow-up ranged from 4.0 to 20.3 years, with a weighted mean (SD) of 7.2 (3.7) years. All studies assessed physical activity through self-reported questionnaires, but the categorisation of physical activity varied across studies. Though there was a slight variation in the degree of covariate adjustment, all but three studies adjusted for established risk factors such as age, sex, BMI, smoking, alcohol consumption, prevalent hypertension, prevalent diabetes, and prevalent CHD. Fifteen studies were at moderate risk of bias and 8 were at serious risk of bias (Appendix 4).

**Table 1:** Baseline characteristics of observational cohort studies included in review

Author, year of publication	Study name	Country	Baseline year	Mean/median age (yrs)	Average BMI (kg/m^2^)	Males (%)	Average follow-up (yrs)	PA exposure	No. of AF cases	No. of participants	Covariates adjusted for
Frost 2005	DDCH	Denmark	1993–1997	55.1	26.3	51.0	5.7	Work-related PA	418	38,400	Age, body height, BMI, smoking, consumption of alcohol, SBP, treatment for hypertension, total serum cholesterol, duration of sporting activities, and level of education
Mozaffarian 2008	CHS	USA	1989–1990	73.0	27.0	42.0	12.0	Leisure-time PA	1061	5446	Age, gender, race, enrolment site, education, smoking status, pack-years of smoking, CHD, chronic pulmonary disease, DM, alcohol use, and beta-blocker use
Aizer 2009	PHS	USA	1982	NR	NR	100.0	12.0	Vigorous exercise	1661	16,921	Age, treatment assignment (aspirin or placebo, beta carotene or placebo), BMI, DM, history of hypertension, history of hyperlipidemia, parental history of premature MI, alcohol intake, smoking habits, fish consumption, multivitamin intake, vitamin C intake, vitamin E intake, LVF, CHF, and evidence of cardiovascular disease
Everett 2011	WHS	USA	1993–2004	54.6	NR	0	14.4	Total leisure-time PA	968	34,759	Age, randomized treatment, cholesterol, current smoking, past smoking, alcohol, DM, race, hypertension and BMI
Thelle 2013	NorPD	Norway	1985–1999	41.4	25.0	47.6	~ 4.0	Leisure-time PA	863	309,540	Age 2005, year of screening, education, BMI, height, daily smoking, self-reported CVD at screening, and dispensed cardiovascular drugs in 2006
Azarbal 2014	WHI	USA	1994–1998	63.4	NR	0	11.5	Total PA	9792	81,317	Age, race, education, BMI, hypertension, DM, hyperlipidemia, CAD, HF, PAD, smoking
Drca 2014	N/A	Sweden	1997–1998	60.0	26.0	100.0	12.0	Leisure-time exercise	4568	44,410	Age, education, smoking status and pack years of smoking, BMI, DM, history of hypertension, history of CHD or HF, family history of MI, aspirin use, and alcohol consumption
Ghorbani 2014	HPFS	USA	2002	68.0	NR	100.0	8.2	Total PA	782	28,169	Age, alcohol intake, smoking, pulmonary disease, aspirin use, omega 3 intake, caffeine intake, and health seeking behaviour index
Huxley 2014	ARIC	USA	1987–1989	54.2	27.8	45.3	18.2	Total PA level	1775	14,219	Age, race, sex, study site, education, income, prior CVD, cigarette smoking, height, and alcohol consumption
Knuiman 2014	BHS	Australia	1994–1995	52.0	26.0	43.6	15.0	Vigorous exercise	343	4267	Age, sex, height, hypertension treatment and BMI terms
Drca 2015	SMC	Sweden	1997	60.0	25.0	0	12.0	Leisure-time PA	2915	36,513	Age, education, smoking status and pack-years of smoking, BMI, DM, history of hypertension, history of CHD or HF, family history of MI, and alcohol consumption
Morseth 2016	Tromso Study	Norway	1986–1987	NR	NR	50.3	20.0	Leisure-time PA	750	20,484	Age, sex, BMI, height, daily smoking, CVD, SBP, DBP, DM, and hypertension treatment
Skielboe 2016	CCHS	Denmark	1976–1978	48.0	25.5	42.2	20.3	Leisure-time PA	1192	17,196	Age, height, BMI, sex, smoking, drinking habits, school education, BP, resting heart rate, spirometry, cardiac medication, DM, IHD and enrolment number
Albrecht 2018	Rotterdam Study	Netherlands	1997–2001	69.4	27.0	41.8	12.6	Total PA	800	7018	Age, sex, other PA types, smoking, previous CVD, alcohol consumption, diet, education
Di Benedetto 2018	EPIC Norfolk	UK	1993–1998	58.5	26.3	45.2	17.1	Total PA	2155	21,499	Age and sex
Garg 2018	REGARDS	USA	2003–2007	63.0	29.0	43.0	9.4	Total PA	725	9576	Age, sex, race, education, income, and geographic region, LVH, alcohol use, CHD, and stroke
Garnvik 2018	HUNT3	Norway	2006–2008	51.5	27.2	45.9	8.1	Leisure-time PA	1459	43,602	Age, sex, current smoking, alcohol use, self-reported CVD, occupational status, BMI
Ogunmoroti 2018	MESA	USA	2000–2003	62.0	28.3	47.0	11.2	Total PA	709	6506	Age, sex, race/ethnicity, education, income and health insurance
Choi 2019	Ansung-Ansan cohort	Korea	2001–2002	50.0	24.5	48.1	11.6	Total PA	167	8,811	Age, sex, residence, education, BMI, comorbidity, alcohol, smoking, and RHR
Hamada 2019	Ningen Dock	Japan	2008–2014	52.4	22.8	75.5	7.0	Total PA	349	65,984	Age and sex
Jin 2019	NHIS	Korea	2002–2013	47.6	23.7	50.0	4.0	Total PA	3443	501,690	Age, sex, BMI, HF, hypertension, DM, previous MI, prior stroke or transient ischemic attack, CKD, smoking, and alcohol drinking
Lee 2019	Kangbuk Samsung Health Study	Korea	2002–2014	37.8	23.4	61.3	5.6	Total PA	304	211,992	Age, sex, center, year of screening examination, smoking status, alcohol intake, education level, BMI, DM, hypertension, CVD, and hs-CRP
Elliot 2020	UK Biobank	UK	2007–2010	56.5	27.3	47.6	7.0	Total PA	8640 (1,266 VAs)	402,406	Age, sex, BMI, smoking, alcohol intake, prevalent Type 2 diabetes, hypertension, sleep apnoea, HF, valvular disease, and CHD

*AF* atrial fibrillation; *BMI* body mass index; *CAD* coronary artery disease; *CHF* congestive heart failure; *CKD* chronic kidney disease; *CVD* cardiovascular disease; *DBP* diastolic blood pressure; *DM* diabetes mellitus; *HF* heart failure; *hs-CRP* high sensitivity C-reactive protein; *IHD* ischemic heart disease; *LVH* left ventricular hypertrophy; *MI* myocardial infarction; *NR* not reported; *PA* physical activity; *PAD* peripheral artery disease; *RHR* resting heart rate; SBP, systolic blood pressure

Study abbreviations: *ARIC* Atherosclerosis Risk in Communities Study; *BHS* Busselton Health Study; *CCHS* Copenhagen City Heart Study; *CHS* Cardiovascular Health Study; *DDCH* Danish Diet, Cancer, and Health study; *EPIC* European Prospective Investigation into Cancer and Nutrition; *HPFS* Health Professional Follow-up Study; *MESA* Multi-Ethnic Study of Atherosclerosis; *NHIS* National Health Insurance Service; *NorPD* Norwegian Prescription Database; *PHS* Physicians Health Study; *REGARDS* Reasons for Geographic and Racial Differences in Stroke; *SMC* Swedish Mammography Cohort; *WHS* Women’s Health Study; *WHI* Women’s Health Initiative

### Physical activity and risk of AF

**Fig. 2:**
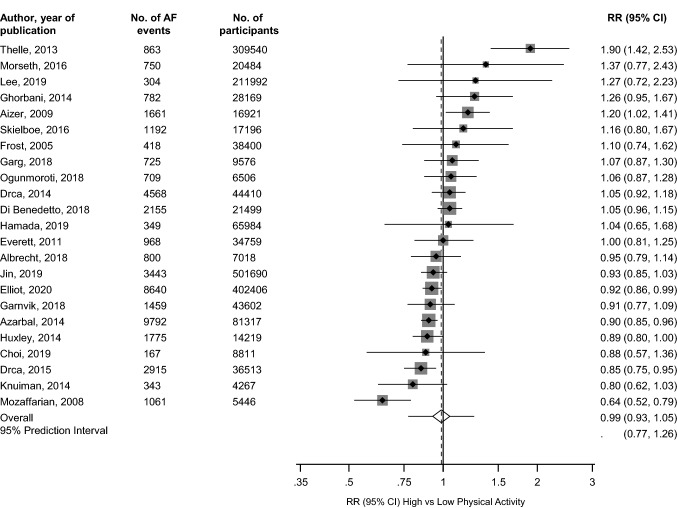
Observational cohort studies of physical activity and risk of atrial fibrillation included in meta-analysis. The summary estimate presented was calculated using random effects models and was based on fully adjusted estimates; sizes of data markers are proportional to the inverse of the variance of the relative ratio; *AF* atrial fibrillation; *CI* confidence interval (bars); *PA* physical activity; *RR* relative risk

**Fig. 3:**
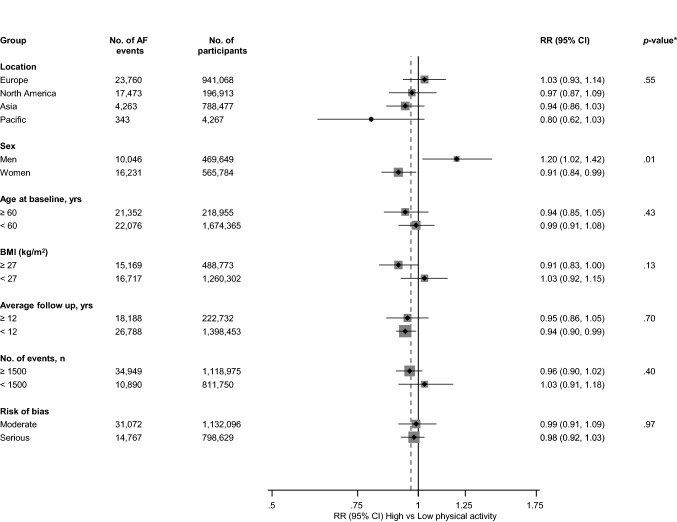
Relative risks for atrial fibrillation comparing maximal versus minimal amount of physical activity, grouped according to several study-level characteristics. The summary estimates presented were calculated using random effects models; *AF* atrial fibrillation; *BMI* body mass index; *CI* confidence interval (bars); *PA* physical activity; *RR* relative risk; *p-value for meta-regression

The pooled multivariable-adjusted RR (95% CI) of AF comparing the most physically active versus the least physically active groups was 0.99 (0.93–1.05) (Fig. 2). The 95% prediction interval for the pooled RR was 0.77–1.26%, suggesting that the true RR for any new single study will usually fall within this range. There was substantial heterogeneity between the contributing studies (*I*^*2*^ = 70%, 54 to 80%; *p* < 0.001), which was partly explained by sex (*p*-value for meta-regression = 0.01) (Fig. 3). Regular physical activity was associated with an increased risk of AF in men RR (95% CI) of 1.20 (1.02–1.42) and a decreased risk of AF in women RR (95% CI) of 0.91 (0.84–0.99).

### Publication bias

A funnel plot of the 23 studies reporting on the associations between physical activity and risk of AF showed no evidence of asymmetry (Appendix 5), which was consistent with Egger’s regression symmetry test (*p* = 0.08). We found no evidence of such selective reporting when studies were grouped by size in meta-regression analysis (Fig. 3).

### GRADE summary of findings

GRADE ratings for the overall population and that in men and women were assessed and are reported in Appendix 6. GRADE quality of the evidence ranged from low to moderate.

### Comments

#### Summary of main findings

Given the controversial relationship between physical activity and the risk of AF and the inclusion of case–control designs and a mix of general population participants and athletes in previous pooled analyses, we evaluated the relationship by conducting a meta-analysis of only population-based observational cohort studies limited to general populations. In a pooled analysis of 23 unique cohort studies comprising of approximately 2 million participants, there was no strong evidence suggesting regular physical activity was associated with the risk of AF in the overall population. However, a subgroup analysis by sex demonstrated that regular physical activity was associated with an increased risk of AF in men and a decreased risk in women. The quality of the evidence ranged from low to moderate.

### Comparison with previous work

There have been several previous efforts to aggregate the existing data on the relationship between physical activity and AF and the findings have mostly been divergent. Our overall null findings are consistent with previous reviews. In a pooled analysis of four prospective cohort studies based on general populations, findings of Ofman and colleagues did not support a significant association between regular physical activity and increased or decreased risk of AF [16]. In an analysis of 10 studies based on the general population, Kwok and colleagues demonstrated no evidence of an association between intensive or leisure-time activity and the risk of AF [15]. Ricci and colleagues in a pooled random-effects meta-analysis of 18 studies showed no significant association between physical activity and risk of AF, though a dose–response analysis suggested a J-shaped relationship [17]. A significant limitation of their dose–response analysis, as reported by the authors themselves, was that it was only based on a subset of studies because of limited data on studies reporting physical activity exposure in terms of metabolic equivalents. As described by Orsini et al. [54] a dose–response analysis requires that the number of cases, person-years of follow-up or non-cases, and the RRs with the variance estimates for at least three quantitative categories of exposure levels are known for each study. Our attempt to extract these data yielded complete data for only three studies, which was not enough information to evaluate the dose-response relationship. In an attempt to address the specificity of the association, we conducted subgroup analyses by several relevant study-level characteristics, including age, sex and BMI, and demonstrated there was evidence of effect modification by sex. Several reviews have also reported on the sex-specific associations of physical activity with AF. Mohanty and colleagues reported that moderate amounts of physical activity reduced the risk of AF in both men and women, but observed that intense exercise had a sex-specific association with AF risk. [19] Zhu and colleagues, in their evaluation of sex differences in the association, showed total physical activity to be associated with an increased AF risk in men and a reduced risk in women. [18] The major limitations of some of these previous reviews were the inclusion of case–control designs and a mix of general population participants and professional athletes. [17, 19] Our findings are based on the most up to date evidence on general population participants and limited to cohort study designs, which are next to randomised controlled trials in the hierarchy of evidence pyramid.

### Possible explanations for findings

Given the well-established link between physical activity and CVD outcomes and the fact that cardiac arrhythmias and other cardiovascular outcomes share common risk factors, some of the findings may seem unexpected. The current results, however, do suggest that sex is a potential effect modifier of the association between physical activity and AF and which could be driving the overall null associations observed in previous studies and the current one. There may be important differences between males and females in the pathophysiology of AF attributed to physical activity. It is generally reported that vigorous- or high-intensity physical activities (HIPAs) might be associated with potential toxic effects such as cardiac dysfunction and arrhythmias, especially in endurance sports such as marathons and cycling races [55, 56]. The suggested physiological mechanisms for this observation include increased vagal tone, bradycardia, increased volume load and stretch, atrial fibrosis and inflammation [57, 58]. The reasons for the contrasting effect of physical activity on AF risk in males and females are unclear, but there have been suggestions. Sex differences in cardiac adaptation to physical activity have been proposed [18]. Females compared to males, have less unfavourable cardiac structural remodelling after a comparable amount of physical activity [59]. Other factors implicated include comorbidities, amount and intensity of physical activity, the impact of sex hormones, pro-inflammatory status and autonomic tone. The lower AF risk observed in females relative to males may be attributed to fewer comorbidities, amplified inflammation cascade, deeper vagal enhancement (lower sympathetic tone), and less amount and intensity of physical activity [59–62]. Finally, the sex-specific differences may just be a chance finding and not due to true differences.

### Implications of findings

There are potential clinical implications concerning our current findings. Though vigorous- or high-intensity endurance sports might cause potential toxic effects such as arrhythmias, the evidence has not been consistent or strong enough to recommend avoiding these activities. [55] The current findings suggest that regular or high levels of physical activity may be associated with increased AF in men. However, the amount, intensity, and duration of physical activity beyond which the risk of AF is increased needs clarification. The evidence on the vascular health and mortality benefits attributed to regular physical activity is overwhelming. Even for HIPA regimens whose intensities and volumes far exceed those proposed by guideline recommendations, [63] their long-term protective effects on mortality by far outweigh their risk of adverse events such as AF. [64] Furthermore, there is a potential for physical activity to reduce the risk of death and symptoms of AF among veteran endurance athletes with AF. [65] Physical activity should be promoted given its beneficial effect on overall health and mental well-being as well as mortality reduction. The Physical Activity Guidelines Advisory Committee Scientific Report recommends 150–300 min/week of moderate-intensity or 75–150 min/week of vigorous-intensity aerobic PA/exercise for adults, as these levels are associated with substantial benefits in the majority of people. [66].

### Strengths and limitations

The strengths of this review are the inclusion of only observational cohort designs, exploration of sources of heterogeneity using stratification by several study level characteristics and evaluating for small study effects. We also assessed the risk of bias and the quality of the evidence using validated and well-established tools. The limitations were mostly inherent to the included studies. A major limitation was the inability to fully examine the impact of consistent adjustment for potential confounding factors, because the review was based on variably adjusted data reported in the published literature. However, the majority of included studies adjusted for established risk factors such as age, sex, BMI, smoking, alcohol consumption, prevalent hypertension, prevalent diabetes, and prevalent CHD. There was variance in the assessment, definition and categorisation of the physical activity exposure by the studies, which precluded transformation into consistent comparisons; hence comparisons could only be made between the most and least active. Whether the association varied by the type of physical activity (aerobic vs. resistance) could not be evaluated because the majority of studies reported physical activity as a combination of the two types. Physical activity was self-reported and hence the potential for misclassification bias. Data extraction could not be performed in pairs due to manpower resource constraints. Hence, one author initially extracted the data, which was independently checked by a second author with disagreements resolved with the involvement of a third author. To ensure completeness, the corresponding author also double-checked the extracted data. Given all the limitations, the findings should be interpreted with caution. We propose an individual participant data meta-analysis of these observational cohort studies to address issues with standardization of physical activity and exploration of dose–response relationships.

## Conclusion

New evidence based on a comprehensive meta-analysis of all observational cohort studies suggest that the absence of associations reported between regular physical activity and AF risk in previous general population studies and their aggregate analyses could be driven by sex-differences in the associations—an increased risk in men and a decreased risk in women.

## Supplementary information

Supplementary file1 (DOCX 175kb)

## References

[1] Ruddox V, Sandven I, Munkhaugen J, Skattebu J, Edvardsen T, Otterstad JE (2017). Atrial fibrillation and the risk for myocardial infarction, all-cause mortality and heart failure: a systematic review and meta-analysis. Eur J Prev Cardiol.

[2] Thrall G, Lane D, Carroll D, Lip GY (2006). Quality of life in patients with atrial fibrillation: a systematic review. Am J Med..

[3] Krahn AD, Manfreda J, Tate RB, Mathewson FA, Cuddy TE (1995). The natural history of atrial fibrillation: incidence, risk factors, and prognosis in the Manitoba follow-up study. Am J Med.

[4] Cheng W, Zhang Z, Cheng W, Yang C, Diao L, Liu W (2018). Associations of leisure-time physical activity with cardiovascular mortality: a systematic review and meta-analysis of 44 prospective cohort studies. Eur J Prev Cardiol.

[5] Lear SA, Hu W, Rangarajan S (2017). The effect of physical activity on mortality and cardiovascular disease in 130 000 people from 17 high-income, middle-income, and low-income countries: the PURE study. Lancet.

[6] Kyu HH, Bachman VF, Alexander LT (2016). Physical activity and risk of breast cancer, colon cancer, diabetes, ischemic heart disease, and ischemic stroke events: systematic review and dose-response meta-analysis for the global burden of disease study 2013. BMJ.

[7] Sesso HD, Paffenbarger RS, Lee IM (2000). Physical activity and coronary heart disease in men: the harvard alumni health study. Circulation.

[8] Elliott AD, Linz D, Mishima R (2020). Association between physical activity and risk of incident arrhythmias in 402 406 individuals: evidence from the UK Biobank cohort. Eur Heart J.

[9] Mozaffarian D, Furberg CD, Psaty BM, Siscovick D (2008). Physical activity and incidence of atrial fibrillation in older adults: the cardiovascular health study. Circulation.

[10] Drca N, Wolk A, Jensen-Urstad M, Larsson SC (2015). Physical activity is associated with a reduced risk of atrial fibrillation in middle-aged and elderly women. Heart.

[11] Aizer A, Gaziano JM, Cook NR, Manson JE, Buring JE, Albert CM (2009). Relation of vigorous exercise to risk of atrial fibrillation. Am J Cardiol.

[12] Thelle DS, Selmer R, Gjesdal K (2013). Resting heart rate and physical activity as risk factors for lone atrial fibrillation: a prospective study of 309,540 men and women. Heart.

[13] Jin MN, Yang PS, Song C (2019). Physical activity and risk of atrial fibrillation: a nationwide cohort study in general population. Sci Rep.

[14] Bapat A, Zhang Y, Post WS (2015). Relation of physical activity and incident atrial fibrillation (from the multi-ethnic study of atherosclerosis). Am J Cardiol.

[15] Kwok CS, Anderson SG, Myint PK, Mamas MA, Loke YK (2014). Physical activity and incidence of atrial fibrillation: a systematic review and meta-analysis. Int J Cardiol.

[16] Ofman P, Khawaja O, Rahilly-Tierney CR (2013). Regular physical activity and risk of atrial fibrillation: a systematic review and meta-analysis. Circ Arrhythm Electrophysiol.

[17] Ricci C, Gervasi F, Gaeta M, Smuts CM, Schutte AE, Leitzmann MF (2018). Physical activity volume in relation to risk of atrial fibrillation. A non-linear meta-regression analysis. Eur J Prev Cardiol.

[18] Zhu WG, Wan R, Din Y, Xu Z, Yang X, Hong K (2016). Sex differences in the association between regular physical activity and incident atrial fibrillation: a meta-analysis of 13 prospective studies. Clin Cardiol.

[19] Mohanty S, Mohanty P, Tamaki M (2016). Differential association of exercise intensity with risk of atrial fibrillation in men and women: evidence from a meta-analysis. J Cardiovasc Electrophysiol.

[20] Moher D, Liberati A, Tetzlaff J, Altman DG (2009). Preferred reporting items for systematic reviews and meta-analyses: the PRISMA statement. PLoS Med.

[21] Stroup DF, Berlin JA, Morton SC (2000). Meta-analysis of observational studies in epidemiology. JAMA J Am Med Assoc.

[22] Sterne JA, Hernan MA, Reeves BC (2016). ROBINS-I: a tool for assessing risk of bias in non-randomised studies of interventions. BMJ.

[23] Guyatt G, Oxman AD, Akl EA (2011). GRADE guidelines: 1. Introduction-GRADE evidence profiles and summary of findings tables. J Clin Epidemiol..

[24] Hamada R, Muto S (2019). Simple risk model and score for predicting of incident atrial fibrillation in Japanese. J Cardiol.

[25] Kunutsor SK, Apekey TA, Cheung BM (2015). Gamma-glutamyltransferase and risk of hypertension: a systematic review and dose-response meta-analysis of prospective evidence. J Hypertens.

[26] Kunutsor SK, Apekey TA, Khan H (2014). Liver enzymes and risk of cardiovascular disease in the general population: a meta-analysis of prospective cohort studies. Atherosclerosis.

[27] Kunutsor SK, Makikallio TH, Seidu S (2020). Physical activity and risk of venous thromboembolism: systematic review and meta-analysis of prospective cohort studies. Eur J Epidemiol.

[28] Chen HG, Sheng LT, Zhang YB (2019). Association of vitamin K with cardiovascular events and all-cause mortality: a systematic review and meta-analysis. Eur J Nutr.

[29] Greenland S, Longnecker MP (1992). Methods for trend estimation from summarized dose-response data, with applications to meta-analysis. Am J Epidemiol.

[30] R DerSimonian N Laird. Meta-analysis in clinical trials Control Clin Trials. 1986;7:3 177–188. 0197-2456(86)90046-2[pii]. 10.1016/0197-2456(86)90046-2 3802833

[31] Higgins JP, Thompson SG, Deeks JJ, Altman DG (2003). Measuring inconsistency in meta-analyses. BMJ.

[32] Higgins JP, Thompson SG (2002). Quantifying heterogeneity in a meta-analysis. Stat Med.

[33] Riley RD, Higgins JP, Deeks JJ (2011). Interpretation of random effects meta-analyses. BMJ.

[34] Higgins JP, Thompson SG, Spiegelhalter DJ (2009). A re-evaluation of random-effects meta-analysis. J R Stat Soc Ser A Stat Soc.

[35] Thompson SG, Sharp SJ (1999). Explaining heterogeneity in meta-analysis: a comparison of methods. Stat Med.

[36] Begg CB, Mazumdar M (1994). Operating characteristics of a rank correlation test for publication bias. Biometrics.

[37] Egger M, Davey Smith G, Schneider M, Minder C (1997). Bias in meta-analysis detected by a simple, graphical test. BMJ.

[38] Ogunmoroti O, Michos ED, Aronis KN (2018). Life's simple 7 and the risk of atrial fibrillation: the multi-ethnic study of atherosclerosis. Atherosclerosis.

[39] Everett BM, Conen D, Buring JE, Moorthy MV, Lee IM, Albert CM (2011). Physical activity and the risk of incident atrial fibrillation in women. Circ Cardiovasc Qual Outcomes.

[40] Azarbal F, Stefanick ML, Salmoirago-Blotcher E (2014). Obesity, physical activity, and their interaction in incident atrial fibrillation in postmenopausal women. J Am Heart Assoc.

[41] Huxley RR, Misialek JR, Agarwal SK (2014). Physical activity, obesity, weight change, and risk of atrial fibrillation: the atherosclerosis risk in communities study. Circ Arrhythm Electrophysiol.

[42] Drca N, Wolk A, Jensen-Urstad M, Larsson SC (2014). Atrial fibrillation is associated with different levels of physical activity levels at different ages in men. Heart.

[43] Morseth B, Graff-Iversen S, Jacobsen BK (2016). Physical activity, resting heart rate, and atrial fibrillation: the Tromso Study. Eur Heart J.

[44] Skielboe AK, Marott JL, Dixen U, Friberg JB, Jensen GB (2016). Occupational physical activity, but not leisure-time physical activity increases the risk of atrial fibrillation: The Copenhagen City Heart Study. Eur J Prev Cardiol.

[45] Albrecht M, Koolhaas CM, Schoufour JD (2018). Physical activity types and atrial fibrillation risk in the middle-aged and elderly: the Rotterdam study. Eur J Prev Cardiol.

[46] Garg PK, O'Neal WT, Ogunsua A (2018). Usefulness of the American heart association's life simple 7 to predict the risk of atrial fibrillation (from the reasons for geographic and racial differences in stroke [REGARDS] study). Am J Cardiol.

[47] Garnvik LE, Malmo V, Janszky I, Wisloff U, Loennechen JP, Nes BM (2018). Physical activity modifies the risk of atrial fibrillation in obese individuals: the HUNT3 study. Eur J Prev Cardiol.

[48] Di Benedetto L, Michels G, Luben R, Khaw KT, Pfister R (2018). Individual and combined impact of lifestyle factors on atrial fibrillation in apparently healthy men and women: the EPIC-Norfolk prospective population study. Eur J Prev Cardiol.

[49] Ghorbani A, Willett WC, Mozaffarian D (2014). Physical activity and incidence of atrial fibrillation: the health professionals follow-up study. Circulation.

[50] Choi YW, Park M, Lim YH (2019). Independent effect of physical activity and resting heart rate on the incidence of atrial fibrillation in the general population. Sci Rep.

[51] Lee SH, Ryu S, Lee JY, Seo DC, Kim BJ, Sung KC (2019). Association between self-reported physical activity and incident atrial fibrillation in a young Korean population. Sci Rep.

[52] Knuiman M, Briffa T, Divitini M (2014). A cohort study examination of established and emerging risk factors for atrial fibrillation: the Busselton Health Study. Eur J Epidemiol.

[53] Frost L, Frost P, Vestergaard P (2005). Work related physical activity and risk of a hospital discharge diagnosis of atrial fibrillation or flutter: the Danish diet, cancer, and health study. Occup Environ Med.

[54] Orsini N, Bellocco R, Greenland S (2006). Generalized least squares for trend estimation of summarized dose-response data. Stata J.

[55] Lavie CJ, Arena R, Swift DL (2015). Exercise and the cardiovascular system: clinical science and cardiovascular outcomes. Circ Res.

[56] Laukkanen JA, Kunutsor SK, Ozemek C (2019). Cross-country skiing and running's association with cardiovascular events and all-cause mortality: a review of the evidence. Prog Cardiovasc Dis.

[57] Mont L, Elosua R, Brugada J (2009). Endurance sport practice as a risk factor for atrial fibrillation and atrial flutter. Europace.

[58] Bosomworth NJ (2015). Atrial fibrillation and physical activity: Should we exercise caution?. Can Fam Phys.

[59] Wilhelm M, Roten L, Tanner H, Wilhelm I, Schmid JP, Saner H (2011). Gender differences of atrial and ventricular remodeling and autonomic tone in nonelite athletes. Am J Cardiol.

[60] Gill SK, Teixeira A, Rama L (2015). Circulatory endotoxin concentration and cytokine profile in response to exertional-heat stress during a multi-stage ultra-marathon competition. Exerc Immunol Rev.

[61] Thompson PD (2002). Additional steps for cardiovascular health. N Engl J Med.

[62] Myrstad M, Aaronaes M, Graff-Iversen S, Nystad W, Ranhoff AH (2015). Does endurance exercise cause atrial fibrillation in women?. Int J Cardiol.

[63] Brigham and Women's Hospital. Vitamin D and Omega-3 trial (VITAL). In: ClinicalTrials.gov [Internet]. Bethesda (MD): National Library of Medicine (US). 2000- [cited 2012 Nov 20]. Available from: http://www.clinicaltrials.gov/ct2/show/NCT01169259?term=VITAL&rank=1 NLM Identifier: NCT01169259.

[64] Farahmand B, Hållmarker U, Brobert GP, Ahlbom A (2007). Acute mortality during long-distance ski races (Vasaloppet). Scand J Med Sci Sports.

[65] Myrstad M, Aaronaes M, Graff-Iversen S, Ariansen I, Nystad W, Ranhoff AH (2016). Physical activity, symptoms, medication and subjective health among veteran endurance athletes with atrial fibrillation. Clin Res Cardiol.

[66] Piercy KL, Troiano RP, Ballard RM (2018). The physical activity guidelines for Americans. JAMA.

