# Supplementary material for: Physical activity and risk of atrial fibrillation in the general population: meta-analysis of 23 cohort studies involving about 2 million participants
Source: Eur J Epidemiol. 2021 Jan 25;36(3):259–74. doi: 10.1007/s10654-020-00714-4 (PMC8032592; doi:10.1007/s10654-020-00714-4)
Supplement: Supplementary file 1 — Supplementary file1 (DOCX 175kb) [file 10654_2020_714_MOESM1_ESM.docx]

**Supplementary Material**

| **Appendix 1** | PRISMA checklist |
| --- | --- |
| **Appendix 2** | MOOSE checklist |
| **Appendix 3** | Literature search strategy |
| **Appendix 4** | Risk of bias assessment |
| **Appendix 5** | Assessment of small study effects by funnel plot and Egger’s regression symmetry test |
| **Appendix 6** | GRADE summary of findings |

**Appendix 1.** PRISMA checklist

| **Section/topic** | **Item No** | **Checklist item** | **Reported on page No** |
| --- | --- | --- | --- |
| **Title** | | | |
| Title | 1 | Identify the report as a systematic review, meta-analysis, or both | 1 |
| **Abstract** | | | |
| Structured summary | 2 | Provide a structured summary including, as applicable, background, objectives, data sources, study eligibility criteria, participants, interventions, study appraisal and synthesis methods, results, limitations, conclusions and implications of key findings, systematic review registration number | 2 |
| **Introduction** | | | |
| Rationale | 3 | Describe the rationale for the review in the context of what is already known | Introduction |
| Objectives | 4 | Provide an explicit statement of questions being addressed with reference to participants, interventions, comparisons, outcomes, and study design (PICOS) | Introduction |
| **Methods** | | | |
| Protocol and registration | 5 | Indicate if a review protocol exists, if and where it can be accessed (such as web address), and, if available, provide registration information including registration number | Methods |
| Eligibility criteria | 6 | Specify study characteristics (such as PICOS, length of follow-up) and report characteristics (such as years considered, language, publication status) used as criteria for eligibility, giving rationale | Methods |
| Information sources | 7 | Describe all information sources (such as databases with dates of coverage, contact with study authors to identify additional studies) in the search and date last searched | Methods |
| Search | 8 | Present full electronic search strategy for at least one database, including any limits used, such that it could be repeated | Appendix 3 |
| Study selection | 9 | State the process for selecting studies (that is, screening, eligibility, included in systematic review, and, if applicable, included in the meta-analysis) | Methods |
| Data collection process | 10 | Describe method of data extraction from reports (such as piloted forms, independently, in duplicate) and any processes for obtaining and confirming data from investigators | Methods |
| Data items | 11 | List and define all variables for which data were sought (such as PICOS, funding sources) and any assumptions and simplifications made | Methods |
| Risk of bias in individual studies | 12 | Describe methods used for assessing risk of bias of individual studies (including specification of whether this was done at the study or outcome level), and how this information is to be used in any data synthesis | Methods |
| Summary measures | 13 | State the principal summary measures (such as risk ratio, difference in means). | Methods |
| Synthesis of results | 14 | Describe the methods of handling data and combining results of studies, if done, including measures of consistency (such as I^2^ statistic) for each meta-analysis | Methods |
| Risk of bias across studies | 15 | Specify any assessment of risk of bias that may affect the cumulative evidence (such as publication bias, selective reporting within studies) | Methods |
| Additional analyses | 16 | Describe methods of additional analyses (such as sensitivity or subgroup analyses, meta-regression), if done, indicating which were pre-specified | Methods |
| **Results** | | | |
| Study selection | 17 | Give numbers of studies screened, assessed for eligibility, and included in the review, with reasons for exclusions at each stage, ideally with a flow diagram | Results and Figure 1 |
| Study characteristics | 18 | For each study, present characteristics for which data were extracted (such as study size, PICOS, follow-up period) and provide the citations | Results, Table 1 |
| Risk of bias within studies | 19 | Present data on risk of bias of each study and, if available, any outcome-level assessment (see item 12). | Results, Table 1 |
| Results of individual studies | 20 | For all outcomes considered (benefits or harms), present for each study (a) simple summary data for each intervention group and (b) effect estimates and confidence intervals, ideally with a forest plot | Results, Figure 2 |
| Synthesis of results | 21 | Present results of each meta-analysis done, including confidence intervals and measures of consistency | Results, Figure 2 |
| Risk of bias across studies | 22 | Present results of any assessment of risk of bias across studies (see item 15) | Table 1, Figure 3 |
| Additional analysis | 23 | Give results of additional analyses, if done (such as sensitivity or subgroup analyses, meta-regression) (see item 16) | Results; Figure 3; Appendix 4 |
| **Discussion** | | | |
| Summary of evidence | 24 | Summarise the main findings including the strength of evidence for each main outcome; consider their relevance to key groups (such as health care providers, users, and policy makers) | Discussion |
| Limitations | 25 | Discuss limitations at study and outcome level (such as risk of bias), and at review level (such as incomplete retrieval of identified research, reporting bias) | Discussion |
| Conclusions | 26 | Provide a general interpretation of the results in the context of other evidence, and implications for future research | Discussion |
| **Funding** | | | |
| Funding | 27 | Describe sources of funding for the systematic review and other support (such as supply of data) and role of funders for the systematic review | Funding section |

**Appendix 2.** MOOSE checklist

**Physical activity and risk of atrial fibrillation in the general population: meta-analysis of 23 cohort studies involving about 2 million participants**

| **Criteria** | | **Brief description of how the criteria were handled in the review** |
| --- | --- | --- |
| **Reporting of background** | |  |
| √ | Problem definition | Evidence on the association between physical activity and atrial fibrillation (AF) is inconsistent |
| √ | Hypothesis statement | Regular physical activity is associated with AF |
| √ | Description of study outcomes | Atrial fibrillation |
| √ | Type of exposure | Physical activity |
| √ | Type of study designs used | Observational cohort studies |
| √ | Study population | Adult general populations with assessment of physical activity at study entry with at least 1 year follow-up |
| **Reporting of search strategy should include** | |  |
| √ | Qualifications of searchers | Setor K. Kunutsor, PhD; Samuel Seidu, MD |
| √ | Search strategy, including time period included in the synthesis and keywords | Time period: from inception to 23 October 2020  The detailed search strategy can be found in Appendix 3 |
| √ | Databases and registries searched | MEDLINE, Embase, Web of Science |
| √ | Search software used, name and version, including special features | OvidSP was used to search Embase and MEDLINE  EndNote X9 used to manage references |
| √ | Use of hand searching | We searched bibliographies of retrieved papers |
| √ | List of citations located and those excluded, including justifications | Details of the literature search process are outlined in the flow chart. The citation list for excluded studies are available on request. |
| √ | Method of addressing articles published in languages other than English | Not applicable |
| √ | Method of handling abstracts and unpublished studies | We included a conference abstract that had been published, but not as a full article |
| √ | Description of any contact with authors | None |
| **Reporting of methods should include** | |  |
| √ | Description of relevance or appropriateness of studies assembled for assessing the hypothesis to be tested | Detailed inclusion and exclusion criteria are described in the Methods section. |
| √ | Rationale for the selection and coding of data | Data extracted from each of the studies were relevant to the population characteristics, study design, exposure, and outcome. |
| √ | Assessment of confounding | We assessed confounding by ranking individual studies on the basis of different adjustment levels and performed sub-group analyses to evaluate differences in the overall estimates according to levels of adjustment. |
| √ | Assessment of study quality, including blinding of quality assessors; stratification or regression on possible predictors of study results | Study quality was assessed based on the nine-star Newcastle–Ottawa Scale using pre-defined criteria namely: population representativeness, comparability (adjustment of confounders), ascertainment of outcome. Sensitivity analyses by several quality indicators such as study size, duration of follow-up, and adjustment factors. |
| √ | Assessment of heterogeneity | Heterogeneity of the studies was quantified with I^2^ statistic that provides the relative amount of variance of the summary effect due to the between-study heterogeneity and explored using meta-regression and stratified analyses |
| √ | Description of statistical methods in sufficient detail to be replicated | Description of methods of meta-analyses, sensitivity analyses, meta-regression and assessment of publication bias are detailed in the methods. We performed random effects meta-analysis with Stata 15. |
| √ | Provision of appropriate tables and graphics | Table 1; Figures 1-3; Appendix 4 |
| **Reporting of results should include** | |  |
| √ | Graph summarizing individual study estimates and overall estimate | Figure 2 |
| √ | Table giving descriptive information for each study included | Table 1 |
| √ | Results of sensitivity testing | Sensitivity analysis was conducted to assess the influence of some large studies and low-quality studies on the pooled estimate. |
| √ | Indication of statistical uncertainty of findings | 95% confidence intervals were presented with all summary estimates, I^2^ values and results of sensitivity analyses |
| **Reporting of discussion should include** | |  |
| √ | Quantitative assessment of bias | Sensitivity analyses indicate heterogeneity in strengths of the association due to most common biases in observational studies. The systematic review is limited in scope, as it involves published data. Individual participant data is needed. Limitations have been discussed. |
| √ | Justification for exclusion | All studies were excluded based on the pre-defined inclusion criteria in methods section. |
| √ | Assessment of quality of included studies | Brief discussion included in ‘Methods’ section |
| **Reporting of conclusions should include** | |  |
| √ | Consideration of alternative explanations for observed results | Discussion |
| √ | Generalization of the conclusions | Discussed in the context of the results. |
| √ | Guidelines for future research | We recommend individual participant data meta-analysis |
| √ | Disclosure of funding source | In “Acknowledgement” section |

**Appendix 3.** Literature search strategy

Relevant studies, published from inception to 23 October 2020 (date last searched), were identified through electronic searches limited to the English language using MEDLINE, Embase, and Web of Science databases. Electronic searches were supplemented by scanning reference lists of articles identified for all relevant studies (including review articles) and by hand searching of relevant journals.

| Database: Ovid MEDLINE(R) <1946 to present>  Search Strategy:  --------------------------------------------------------------------------------  1 exp Atrial Fibrillation/ (56340)  2 exp Arrhythmias, Cardiac/ (210953)  3 physical activity.mp. or exp Exercise/ (265846)  4 exp Exercise/ (198947)  5 cohort studies/ or longitudinal studies/ or follow-up studies/ or prospective studies/ or retrospective studies/ or cohort.ti,ab. or longitudinal.ti,ab. or prospective.ti,ab. or retrospective.ti,ab. (2659169)  6 1 or 2 (210953)  7 3 or 4 (265846)  8 5 and 6 and 7 (328)  9 limit 8 to humans (321)  ***************************  Each part was specifically translated for searching the other databases (Embase and Web of Science) |
| --- |

**Appendix 4.** Risk of bias assessment

**Appendix 5.** Assessment of small study effects by funnel plot and Egger’s regression symmetry test

**Appendix 6.** GRADE summary of findings

**Question**: [Most physically active] compared to [Least physically active] for [atrial fibrillation]

**Setting**: General population

| **Certainty assessment** | | | | | | | **№ of patients** | | **Effect** | | **Certainty** | **Importance** |
| --- | --- | --- | --- | --- | --- | --- | --- | --- | --- | --- | --- | --- |
| **№ of studies** | **Study design** | **Risk of bias** | **Inconsistency** | **Indirectness** | **Imprecision** | **Other considerations** | **[Most physically active]** | **[Least physically active]** | **Relative (95% CI)** | **Absolute (95% CI)** |  |  |
| **AF in overall population (follow up: mean 7.2 years)** | | | | | | | | | | | | |
| 23 | observational studies | not serious | serious ^a^ | not serious | not serious | none |  |  | **RR 0.99** (0.93 to 1.05) | **1 fewer per 1,000** (from 1 fewer to 1 fewer) | ⨁⨁⨁◯ MODERATE |  |
| **AF in men (follow up: mean 8.7 years)** | | | | | | | | | | | | |
| 7 | observational studies | not serious | very serious ^b^ | not serious | not serious | none |  |  | **RR 1.20** (1.02 to 1.42) | **1 fewer per 1,000** (from 1 fewer to 1 fewer) | ⨁⨁◯◯ LOW |  |
| **AF in women (follow up: mean 9.5 years)** | | | | | | | | | | | | |
| 7 | observational studies | not serious | serious ^c^ | not serious | not serious | none |  |  | **RR 0.91** (0.84 to 0.99) | **1 fewer per 1,000** (from 1 fewer to 1 fewer) | ⨁⨁⨁◯ MODERATE |  |

**CI:** Confidence interval; **RR:** Risk ratio

**Explanations**

a. I-squared=70%

b. I-squared=86%

c. I-squared=56%
